# The Effect of Culture-Based Breast Health Education Program on Breast Self-Examination Practice Among High-Risk Women: Randomized Controlled Trial

**DOI:** 10.2196/85340

**Published:** 2026-06-18

**Authors:** Sumaira Naz, Sureeporn Thanasilp, Wasinee Wisesrith

**Affiliations:** 1 Faculty of Nursing, Chulalongkorn University, Borommaratchachonnanisisattaphat, 11th Floor, Rama I Rd, Wang Mai, Pathum Wan, Bangkok, 10330, Thailand, 1 +66-2-218-1131, 1 +66-2-218-1130

**Keywords:** breast neoplasm, breast self-examination, cultural competency, health education, women, randomized controlled trial.

## Abstract

**Background:**

Breast cancer (BC) symptom awareness through screening measures, such as breast self-examination practice (BSEP), is promoted in low-resource countries. The Muslim culture has a unique impact on family support for women’s health care decisions, especially from the mother. Given the proven effect of educational intervention on breast health through interactive sessions and mobile health, implementation according to Muslim cultural values through family support, especially from the mother, was lacking, and this study was planned.

**Objective:**

This study aimed to determine the effects of a culture-based breast health education program (CBBHEP) on BSEP in high-risk women.

**Methods:**

A single-site randomized controlled trial was conducted in a hospital on 72 high-risk women, aged 20‐50 years, from August 19 to November 29, 2024. The trial was registered prospectively (ID: ISRCTN-39194106). The participants were matched in pairs by age and educational level, and randomly assigned to experimental and control groups. The intervention program was based on social cognitive theory and the cultural identity concept of the PEN-3 model. The program consisted of face-to-face interactive sessions and a health app called a Women’s Health app for promoting BSEP. The experimental group received CBBHEP, whereas the control group received the usual care. BSEP was measured at baseline and 12 weeks using a translated, valid, and reliable Toronto BSE frequency and proficiency of practice scale. Data analysis was performed using 1-way analysis of covariance and paired sample 1-tailed*t* test.

**Results:**

The participants in the experimental group who received CBBHEP for 12 weeks increased their BSEP much better than those in the control group (*F*_1, 69_=58.908, *η*^2^=0.46; *P*<.001). The results also revealed a statistically significant effect of a higher BSEP in the experimental group (*t*_35_=7.21, Cohen *d*=0.88; *P*<.001).

**Conclusions:**

The findings suggest that health care practitioners can apply this program to increase BSEP among high-risk women through their family support, especially from mothers.

## Introduction

Breast cancer (BC) is among the leading causes of death among women globally and the most frequent type of cancer among women [1,2]. Moreover, 15% of women with BC have a family history of BC [3]. Family history increases the risk of BC, especially if there are close relatives with the disease. If a woman has a mother, sister, or daughter with BC, her chances of developing the condition almost double [4,5].

The highest incidence of BC in Asia has been reported in Pakistani Muslim women (23.1%) [6,7]. There was a family history of BC in 23.8% of Pakistani women. The relative most affected by BC was the mother (47.6%) [8]. The breast self-examination practice (BSEP) notifies women to examine their breasts physically and visually shortly after their periods or at the same time every month to look for any abnormalities [9]. The first and most crucial step in empowering women through the BSEP was to encourage women to actively maintain their health [10]. The early detection of BC symptoms has been positively associated with BSEP [11], which enhances the outcome of treatment [12].

Behavior about BC screening among Pakistani Muslim high-risk women with a family history of the disease revealed that only 15% were aware of BC, and 4.18% were aware of BSE as a BC screening method [13]. BSEP was conducted occasionally by 3.6% of high-risk Pakistani women and frequently by just 1% [14]. All over the nation, patients with BC frequently present late at stage III or IV, with high-risk Pakistani women accounting for approximately 35.2% of these delayed cases [15]. Cultural values have a significant influence on Pakistani women’s awareness of breast health issues, as the majority did not engage in BSEP for a variety of reasons, including the taboo against touching oneself, embarrassment about discussing private body parts, or fear of being examined by a doctor [16,17].

Among the best strategies for changing behavior in high-risk women is family support with BSE instructions or methods, demonstrations, information about health risks, and behavioral stimuli or prompts [18]. Culturally based interventions have proven to be the most successful in promoting health-seeking behaviors [19]. Family is a major unit in Pakistan’s collectivistic Muslim society, which is bound by cultural values and family norms [20].

**Figure 1.**
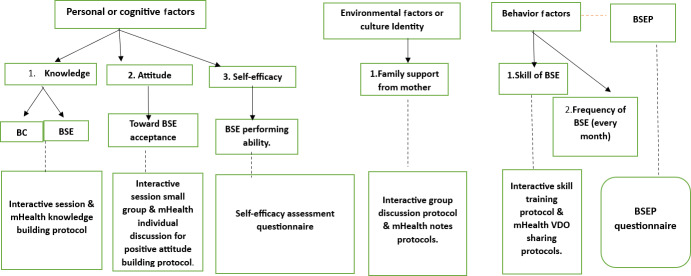
Social cognitive theory and PEN-3 model related to breast self-examination practice (BSEP). BSE: breast self-examination; mHealth: mobile health; VDO: ___.

In this study, based on social cognitive theory (SCT) [21] and the cultural identity concept of the PEN-3 cultural model [22], an intervention was developed, called the Culture-Based Breast Health Education Program (CBBHEP). SCT components, such as personal, environmental, and behavioral factors, were already used for the promotion of BSE [23]. The PEN-3 culture model has also already demonstrated how cultural matters in interventions, such as in cancer awareness and screening [24]. The purpose of concept integration of cultural identity of the PEN-3 Culture model [22], into SCT [21], was to incorporate new ideas, such as the focus of the target group, which helps in the implementation of intervention in the cultural context, as the cultural identity domain of PEN-3 highlights the intervention points of entry. In this study, entry occurred at the level of the family, especially mothers, according to the culture. This CBBHEP included personal factors such as knowledge, attitude, self-efficacy, and environmental factors, as well as social support from the mother in a cultural context, such as a private place for women to promote behavior factors such as BSEP (Figure 1).

Culture-based educational intervention was planned according to Pakistani culture, in which family support, especially from the mother, played an essential role in the health care decisions of their daughters. Cultural identity identifies the target group that should be addressed in health education according to the cultural context. The target group comprised high-risk women with a mother diagnosed with BC who received support from their mothers according to Pakistani culture [20]. BSEP was effectively promoted by educational interventions for BC and BSE, which were shared through interactive lectures, videos, group discussions, or mobile health (mHealth)–based apps [25,26]. Additionally, culturally relevant language and skill-focused mHealth intervention techniques are necessary for BSE [27].

This study was conducted on the proven effects of face-to-face interactive educational and mHealth-based app interventions on BSEP [25,26,28-30]. However, there was a lack of cultural values, such as mother support, which is important for behavioral change. This study fills this knowledge gap in the literature. If proven effective, this can be achieved by educating them about BC and its screening measures, fostering positive attitudes and confidence in their capacity to perform BSE, and with their family support, especially mothers’ support, who are concerned about their daughters.

The objectives of the study were:

1. To compare BSEP between high-risk adult women in an experimental group who received a culture-based breast health program at 3 months with a control group who received usual care.
2. To compare BSEP among high-risk adult women before and after participating in a CBBHEP at 3 months.

The goal of the nurse researcher’s project was to enable high-risk women to recognize BC symptoms at an early stage, which would lower complications and increase the overall survival rate of those affected in low-resource Muslim nations.

## Methods

### Study Design

It is a single-blinded, prospective, and parallel randomized controlled trial using baseline and follow-up assessments with two arms, namely the intervention and control groups, to measure the aim of the study, that is, the effect of CBBHEP on BSEP. The experimental group received CBBHEP and usual care for 12 weeks, whereas the control group received usual care only.

### Ethical Considerations

This study was approved by the Institute of Allied Health Sciences Research Ethics Committee at the National University of Medical Sciences with reference number IAHS/WMC/786/008/admin. The trial was registered at the World Health Organization registry site [31], with ID: ISRCTN-39194106.

### Participants, Setting, and Sample Size

The eligible participants were women aged 20‐37 years, able to speak verbally and write in the local Urdu language; their mother had a BC diagnosis and was receiving treatment at the oncology department of the hospital, had a smartphone with internet access, lived with family, were not pregnant, and were willing to take part in the study. Those who had previously participated in the breast awareness program and experienced known cognitive disorders were excluded. The study was conducted in the oncology department of the Hospital, Taxila, Pakistan, from August to November 2024. *F* test for analysis of covariance (ANCOVA) was used to determine the sample size using G. Power statistics, which were based on the effect size determined in a prior study (effect size=0.515) [28], an overall *α*=.01, power of .90, and attrition rate of 20% due to the sensitivity of the topic. An alpha value of .01 was selected to claim stronger evidence to conclude that an effect would be significant [32]. This resulted in a sample size of 72 participants. The intervention group required 36 subjects, and the control group required 36 participants.

### Recruitment, Randomization, and Masking

The subjects were recruited from the oncology department with the help of a research assistant, who already planned follow-up visits for diagnosed patients. With the permission of the department head, during follow-up calls, the research assistant informed them about the BC awareness program for their daughters. Patient consent was already taken by the department for sharing important information so they can be contacted. In addition, a poster on breast health education was placed in the oncology department. All interested participants were screened for eligibility; 72 were eligible and provided signed consent to participate in the study. Participants were also matched on their age and educational levels. Matching was conducted sequentially as participants enrolled in the study. The minimum age was 20 years, and the maximum was 37 years, among the eligible participants. Age groups were matched within an acceptance range of 3 years. The minimum educational level was 10th grade. Levels were 10th grade, 12th grade, and 16th year of education upon graduation. Acceptance for educational matching was 10th grade, with 11th grade and 14th grade years of education considered for graduation.

After enrollment, 72 participants were randomized into 2 groups (1:1 ratio) by the principal investigator into intervention and control groups (36:36). The principal investigator randomly picked from the allocation concealment opaque envelope to assign the first subject to the assigned condition (experiment), and then, following subjects with similar matched pairs were placed in the second condition (control). Baseline assessment was done for each group after the random allocation into the intervention and control groups. The participants were blinded to the intervention and control groups until trial completion. The random assignment procedure adhered to the CONSORT (Consolidated Standards of Reporting Trials) flow diagram [33].

The CONSORT diagram showed the process of enrollment, random assignment into 2 groups, follow-up, and analysis phases of the study.

### Intervention Material

Reciprocal determination and the self-efficacy construct of SCT [21] were used for the creation and implementation of CBBHEP. It offers a thorough examination of the factors that influence behavior change because human behavior is the result of a dynamic interaction between personal, environmental, and behavioral factors [21]. The personal factors were knowledge, attitude, and self-efficacy, while environmental factors, with integration of the cultural identity concept, the PEN-3 culture model [22], and mother support, which leads to behavior toward BSEP (Table 1). The program was developed by the principal investigator and validated by 3 nurse educators, one software developer, and one female oncologist as experts. Experts were selected based on their working experience in oncology or research. An internet app for women’s health was created by a software developer. Before implementation, the principal investigator created learning materials for in-person classes, educational VDOs or multimedia, handouts, and infographics for mobile apps. The CBBHEP consisted of two parts: Part I, face-to-face sessions, and Part II, the mHealth app. In part-I, 3 face-to-face sessions (20‐25 min) were focused on educational as well as training assemblies to increase knowledge about BC & BSE, building a positive attitude and self-efficacy toward BSE through lecture on PowerPoint presentation in a group of 15‐20 women, structured small group discussion with 7‐10 participants and their mothers for 15‐20 minutes, and small group (7‐10 participants) skill demonstration for 5‐10 minutes, individual redemonstration training with mothers for 5‐10 minutes in a private setting, which leads toward BSE practice. Through behavioral capabilities, people learn knowledge and skills, through positive reinforcement from mothers, can handle barriers, through modeling, learn and develop self-efficacy, which is related to actual behavior performance. Part II included an infographic about information on BC and BSE, the importance of BSE, facts of BC, benefits of BSE video for skill demonstration, a monthly reminder for maintaining continuing in BSE behavior, and writing notes on BSE findings every month with supportive words from family members for the duration of 12 weeks. The mHealth app also helped to improve knowledge, build a positive attitude, and self-efficacy in performing BSE, which ultimately promotes BSE practice in high-risk women. Five experts independently evaluated the quality of the program, its lesson plan and materials, and the mobile app and revised materials after pilot testing on 10% of the required sample. In the pilot testing, interactive lectures, skill training, and small-group discussions were conducted with participants. Additionally, the Women’s Health app (WHA) was installed, and their opinions regarding its feasibility were gathered. The pilot testing was conducted before conducting the actual study, and these 10% of participants were not part of the actual study.

**Table 1.** Culture-based breast health education program on components of social cognitive theory and culture identity concept.

SCT^a^ and culture identity concept of the PEN-3 model and components of CBBHEP^b^	Intervention activities
Personal or cognitive factors, including knowledge, attitude, and self-efficacy of skill performance	
Part I: Face-to-face sessions Knowledge Positive attitude Self-efficacy (mastery experience, vicarious experience, verbal persuasion, physiological, and affective responses).	Information about BC^c^ and BSE^d^ through VDO^e^ and lecture through PowerPoint presentation (20‐25 min) in a group of 15‐20 participants. Positive attitude building toward BSE through a handout about facts of BC and BSE, structured discussion about misperceptions about BC, benefits of BSEP^f^ in a small group of 7‐10 participants (15‐20 min). Enhancement of self-efficacy to perform BSE in a small group (7‐10 participants) training on BSE through VDO sharing and demonstration on breast model or simulator (5‐10 min) for mastery experience and redemonstration by individual participants on breast model and simulator for vicarious experience and verbal persuasion (5‐10 min).
Part-II mHealth^g^ app Awareness Positive attitude Self-efficacy	Informative VDO on BC and BSE. The infographic includes information for enhancing knowledge about BC & BSEP. Infographic to develop a positive attitude through facts of BC and BSE, and the benefits of BSEP. VDO demonstration for enhancing the self-efficacy of BSE performance. Writing findings after doing BSE every month (physiological and emotional state)
Environmental factors or cultural identity, including physical environment and family member support. The culture identity concept also explains the context or setting according to cultural values, such as a private setting, and to identify the target to reach participants, such as mothers, to enhance family support, which improves attitude toward BSEP as well	
Part I: Face-to-face session Positive attitude building with family member support. Setting according to cultural context	Provide a physical environment in the form of a private setting to maintain privacy and modesty for participants and their family members. Mother involvement in a small group discussion with 7‐10 participants, to develop a positive attitude toward BSEP about facts of BC, benefits of BSEP (15‐20 min).
Part-II mHealth app Positive attitude building with family member support.	Writing supporting words from a family member every month.
Behavioral factors, including the frequency of BSEP every month at the same time and the skill of performing BSE. Mother support to promote behavior, such as BSEP, in participants	
Part I: Face-to-face session Frequency of BSE Skill of BSE	BSEP every month, through knowledge enhancement about the timing of BSE (5 min). Individual participant’s skill in performing BSE on the breast model (5 min).
Part-II mHealth app Frequency of BSE Skill of BSE	Monthly reminder for BSE at the same time each month. Watching VDO on the app to improve the skill of BSE performance.

a SCT: social cognitive theory.

b CBBHEP: culture-based breast health education program.

c BC: breast cancer.

d BSE: breast self-examination.

e VDO: ___.

f BSEP: breast self-examination practice.

g mHealth: mobile health.

### Usual Care

The control group received usual care only, whereas the experimental group received CBBHEP with usual care. There are no distinct data for high-risk women in hospital settings. BC and its risk factors and treatment options have been discussed as part of usual care through a pamphlet in the local (Urdu) language.

### Data Collection Procedure

The principal investigator gave the intervention to trained research assistants. Assistants were selected because of their experience in oncology, and training was given about the educational process. Intervention was started with the control group on different dates from the experimental group, and follow-ups were conducted to avoid contamination of the subjects. A trained research assistant collected data (for BSEP at baseline and at 12 wk, and for follow-up and self-efficacy as a mediating factor for program monitoring at baseline, 4th, 8th, and 12 wk). The principal investigator, with the help of a trained research assistant, conducted follow-up visits at the 4th, 8th, and 12th weeks of intervention with individual participants (10‐15 min). An IT specialist collected participation feedback on WHA and mother support at the 4th, 8th, and 12th weeks. The IT specialist compiled the feedback from WHA into paper and shared it with the principal investigator for supporting discussion with participants (Table 2).

**Table 2.** Roles and responsibilities of the principal investigator and assistants during the study.

Component	Deliver	Activities
Recruitment	Assistant nurses	Checking for eligibility for the study. Getting consent to participate in the study. Make matched pairs on the age and education level of participants willing to take part in the study. Baseline assessment of participants.
Interactive session for lecture on BC^a^ and BSE^b^.	Principal investigator	PowerPoint presentation (20-25 min) in a group of 15‐20 participants on BC and BSE importance, mainly by the researcher only.
Small group discussion	Principal investigator and assistant nurses.	Structured discussion about misperceptions about BC, benefits of BSEP^c^ in a small group of 7‐10 participants (15‐20 min) by the researcher and assistant.
Skill training	Principal investigator and assistant nurses.	Training of the steps of BSE on a breast model or female dummy (simulator) by the researcher and assistant in a small group of 7‐10 participants (5‐10 min). Redemonstration of individual participants on breast model or simulator (5‐10 min) by assistant.
Mobile app working	Principal investigator and assistant nurses. IT expert	App installation, data entry, feedback submission, and alarm setting by both the researcher and assistant. The IT expert complied with the participation feedback on the 4th, 8th, and 12th weeks.
Follow-up and app monitoring	Principal investigator, assistant nurses and IT expert	At 4th, 8th, and 12th weeks. IT expert check feedback from app and share with principal investigator. Principal investigator discusses app experience with individual participant (10‐15 min). Individual participant self-efficacy monitoring by the nurse assistant (10‐15 min).

a BC: breast cancer.

b BSE: breast self-examination.

c BSEP: breast self-examination practice.

### Measures

The Toronto BSE frequency and proficiency of practice scale [34] was adapted to measure the BSEP. The instrument was modified in terms of translation into the local Pakistani language and pretested on 30 high-risk women, the minimum sample size required. Cronbach α score was .90, indicating good reliability [35]. It is an 11-item scale that was used to assess BSEP in high-risk women using a five-point Likert scale (score 1 to 5, with 5 being a high score). The mean was obtained from the sum of the scores to determine the BSEP scale score. Higher scores and higher means were seen in this study as indicators of higher BSEP.

The instrument was translated into local language (Urdu) through backward translation techniques by Brislin [36] by followed steps; (1) Forward translation of original (English) version to Urdu version done by two experts (one official bilingual expert and one nurse educator with master’s degree in nursing); (2) an assistant professor of nursing (PhD in nursing,) who is fluent in both languages then checked the translated Urdu version for ambiguous or hard-to-understand wordings; (3) after that, two translators (one official translator and one nurse educator with a master’s degree in nursing) translated the Pakistani version backward to English while being “blinded” to the original English version; and (4) the initial version and modified version were reviewed for concept equivalence; an expert panel that involved all the experts in the translation process identified that the two versions of the instruments were equal and comprised no grammatical issues in meaning.

### Program Monitoring

The self-efficacy scale for BSE [37] was used to monitor program effectiveness. Self-efficacy was selected because it acts as a mediating factor as well for promoting BSEP. The instrument was adapted with permission and translated into Pakistani (Urdu) and tested on high-risk Pakistani women [38]. The scale is scored on a range of 0 (cannot do at all) to 50 (moderately can do), to 100 (definitely can do). Self-efficacy monitoring was done by an experienced nurse educator as a nurse assistant. Additional training for measuring self-efficacy was given by the principal investigator. Nurse assistant did a self-efficacy assessment during follow-up at visits 4th, 8th, and 12th week by assessing individual participant BSE skill on the breast model and by filling a self-administered questionnaire. Participants’ self-efficacy score of 50% and above was considered effective for the intervention during follow-up. A total score of 90% and above was considered satisfactory self-efficacy in the performance of BSE among high-risk women at the end of the intervention, taken from empirical evidence [37]. Participants’ adherence to the program was demonstrated through feedback from the mHealth app.

### Statistical Analysis

Eligible participants were randomized into treatment and control groups. In both groups, baseline characteristics were compared using inferential analyses of independent 1-tailed *t* tests and chi-square tests. This study compared gain scores between the experimental and control groups using one-way ANCOVA to investigate the impact of the CBBHEP on BSEP. The statistical significance level was set at *P*<.01, and the mean was used to determine the significant difference between the mean BSEP, between the intervention and control groups at baseline and 12 weeks, and covariance as the pre-test score [39]. Paired sample *t* statistics were used to measure differences in the mean BSEP level within each group’s data at baseline and at the 12-week follow-up. Before the analysis, assumptions for the 1-way ANCOVA and paired 1-tailed *t* tests were computed. There was no missing data in the analysis.

## Results

### Sample Characteristics

**Figure 2.**
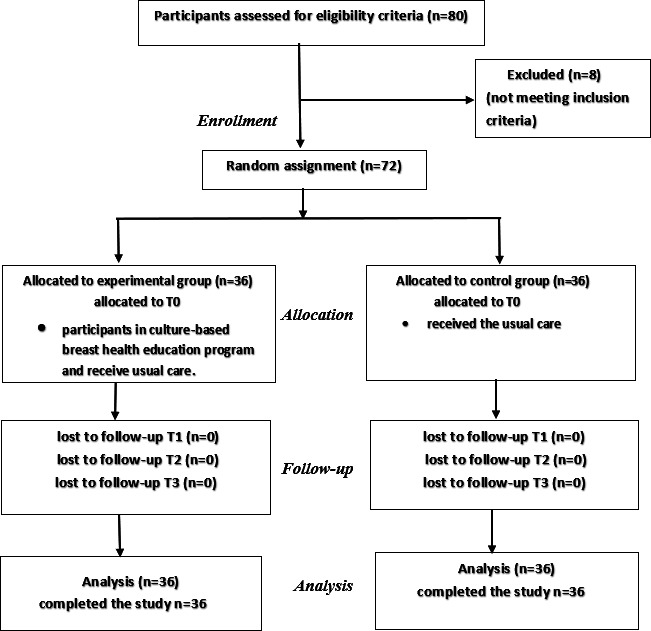
The study CONSORT (Consolidated Standards of Reporting Trials) diagram.

After screening for eligibility, 72 subjects were eventually enrolled in the trial and randomized to one control group (n=36) and one intervention group (n=36; Figure 2). In the 12th week, the retention rate was 100%. At baseline, no discernible demographic differences were observed between groups. The average age was 28 (SD 5.72) years in both groups. The majority of the women in both groups had 10th grade (21/36, 58.3%) education and were mostly married (21/36, 58.3%; Table 3).

**Table 3.** Characteristics of participants.

Characteristics	Intervention group, n (%)	Control group, n (%)	Chi-square (*df*)	*P* value
Age groups (years)			—^a^	—
20-26	16 (44.5)	13 (36.1)		
27-33	13 (36.1)	16 (44.5)		
>33	7 (19.4)	7 (19.4)		
Mean (SD)	28 (5.72)	28 (4.96)		
Educational level			—	—
10th grade	21 (58.3)	21 (58.3)		
12th grade	7 (19.4)	7 (19.4)		
Graduation	8 (22.3)	8 (22.3)		
Occupation history			0.676 (1)	.50
Working	11 (30.5)	10 (27.8)		
Nonworking	25 (69.5)	26 (72.2)		
Marital status			0.963 (1)	.23
Married	21 (58.3)	25 (69.5)		
Un-married	15 (41.7)	11 (30.5)		
Obstetric history (in marriage) having children			1.179 (1)	.55
Yes	14 (66.7)	15 (60)		
No	7 (33.3)	10 (40)		
Did breastfeeding			0.415 (1)	.81
Yes	9 (64.3)	8 (53.4)		
No	5 (35.7)	7 (46.7)		
Menstrual history			0.758 (1)	.28
Regular cycle	30 (83.3)	27 (75)		
Irregular cycle	6 (16.7)	9 (25)		

a Not applicable.

### Intervention Effect on Outcome

The mean was obtained from the sum of the scores obtained to determine the BSEP scale score. The mean (SD) of BSEP at baseline and 12-week measurement in the intervention group and control group were calculated (Table 4).

**Table 4.** Comparison of mean and SD of breast self-examination practice.

BSEP^a^	Mean (SD)		MD^b^
Intervention group			7.93
Baseline	7.17 (4.62)		
12 weeks	15.1 (4.45)		
Control group			0.16
Baseline	7.31 (4.78)		
12 weeks	7.47 (4.92)		

a BSEP: breast self-examination practice.

b MD: mean difference.

The impact of CBBHEP on BSEP between the experimental and control groups was measured using an ANCOVA through gain scores by controlling for the pretest score. This was the difference between the posttest and pretest scores as covariates [39]. The analysis showed that participants who took part in the 12th week of CBBHEP had significantly higher BSEP than those in the control group (*F*_1,69_=58.908; *P*<.001), suggesting that the program had a significant impact on the intervention group’s improvement in BSEP (Table 5).

**Table 5.** One-way analysis of covariance comparison of the mean difference in breast self-examination practice in the intervention and control groups.

Source	SS^a^	*df*	MS^b^	*F* test	*P* value	Partial *η*^2c^
Group	1066.797	1	1066.797	58.908	<.001	.46
Covariate	493.329	1	493.329	27.241	<.001	.28
Error	1249.560	69	18.110	—^d^	—	—

a SS: sum of square.

b MS: mean square.

c Partial *Ƞ*2: effect size.

d Not available.

Paired sample *t* statistics were used to determine the mean difference in BSEP scores at baseline and 12 weeks within the groups (Table 6). The analysis also revealed that the BSEP of the experimental group at baseline and at 12 weeks differed significantly (*t*_35_=7.214; *P*<.001), with BSEP being significantly greater at the 12th week than at baseline. Comparing the pretest and posttest BSEP pairwise for the control group revealed no discernible differences.

**Table 6.** Mean difference of breast self-examination practice score between baseline and 12 weeks within groups.

Variable	Group	*t* test (*df*)		99% CI (LB-UB)	*P* value	Cohen *d* value
BSEP^a^	Experiment	−7.21 (35)		(−10.94 to −4.94)	.<.001	0.88
	Control	−.403 (35)		(−1.29 to 0.95)	.69	0.03

a BSEP: breast self-examination practice.

## Discussion

### Principal Findings

CBBHEP based on components of SCT [21] with integration of culture identity concept of PEN-3 model [22] was found to be effective in promoting BSEP behavior among high-risk women through personal factors such as knowledge of BC and BSE, building positive attitudes toward BSE, and improving the ability to perform BSE with mother support according to their cultural values and local language, as environmental factors concluded over literature support [25-27].

CBBHEP was developed to create awareness about BC and its screening measures, especially BSEP behavior. Pakistani women have many cultural barriers that affect the BC screening behavior, including BSE [16,17,40]. The cultural issues were related to female shyness, modesty, and misbeliefs about breasts as the most sensitive parts of their body that should be concealed or hidden all the time, and it’s only related to sexuality [16]. Embarrassment and an uncomfortable feeling to let practitioners do their breast examination and show it to female doctors for routine check-ups or tests, as it affects their confidentiality [17]. Misconceptions of BC about faith healers can cure the BC, and all lumps would be BC. A significant number also believed BC to be a result of God’s curse or evil eye [41].

The program was conducted in the local (Urdu) language and in a separate room where no men were given access to minimize the cultural barrier related to the confidentiality of women, to reduce shyness, and to maintain female modesty. Mothers of high-risk women were also part of the study because in Pakistani culture, women’s health-related issues are not openly discussed with male members of the family. Mothers were included because of their dominant role, especially for their daughters in families. During the interactive sessions, awareness about BC, screening measures such as BSE, and its importance was given through a PowerPoint presentation, and a VDO about the importance of breast health by religious women was shared to give them motivation and to make them aware that the breast is also like other parts of the body; it’s not only an organ related to sexuality, so breast health is also important for women. During a small discussion group session with mothers and high-risk women, common beliefs and misperceptions about BC were discussed to make their attitude positive for BC screening, especially BSE, by educating them about the facts of BC and the benefits of BSE for breast health awareness. During the training session, a VDO for the BSE skill was shared, and a breast model and simulator were used for demonstration. Redemonstration by each participant was taken on the breast model separately in a private room to maintain privacy and modesty. In WHA, information about BC, BSE, benefits of BSE, and facts about BSE were given for their reading as well, and a VDO on BSE skill was also part of the mHealth app.

According to empirical data, SCT-based educational intervention for promoting BSE was found to be effective [42]. Smartphone apps have been proven to improve BSE performance. A reminder alarm for the intimation of proper time for BSE could have a positive effect on BSEP because people spend a lot of time on their smartphones, and the lessons they learn from these apps, which include educational films, stick in their memory [43]. A smartphone-based educational intervention that included BSE knowledge tasks and monthly reminder messages was found to be 4 times higher BSEP in the intervention group than in the control group [42]. CBBHEP was more effective in promoting BSEP behavior as it was in the local language, including WHA. The PEN-3 model [22] was integrated into SCT [21] as a mother support because it has a potential source to educate and promote BC awareness and prevention among daughters. A mother may affect her daughter’s sense of self-efficacy in overcoming perceived barriers to BC prevention and early detection of BC symptoms in the form of informational support, such as advice or guidance, and by providing emotional support like empathy. Furthermore, exposure to a mother’s attitude and health behavior practices toward BC prevention may influence a daughter’s conception about the benefits and susceptibility of BC prevention. Thus, a mother’s knowledge about family history, modeling norms of healthy lifestyle practices [44], and support of health prevention behaviors could be influential in their daughter’s development of evolving behaviors that contribute to the awareness and prevention measures such as BC screening [45]. The program was acceptable for all participants, and they were motivated to continue the whole program after interactive sessions, as indicated by their feedback through WHA, which also indicates their acceptance of the program as an indication of a 100% retention rate. In the diary notes, participants shared their experiences with BSEP, including the fact that all results were normal, and that supportive remarks from my mother urged me to complete the BSE on time. “My mother reminded me of the BSE time and urged me to teach your sister the skill of doing BSE as well. This increased WHA’s efficacy and fostered a favorable attitude toward BC and BSE behavior. Monthly participant feedback via online data made it easier to understand the role of mothers and the efficacy of the intervention. Participants shared that after participating in this program, mothers’ attitudes were more positive: “She encouraged me to do BSE every month.” “After meeting with you, my mother said, “You must do BSE.” Social support-based educational interventions significantly improved women’s screening behavior for BC [46]. Thus, smartphone apps have been proven to improve BSE performance, but social support will close the gap toward behavior, and eventually accelerate behavior change. Studies show that social support plays a key role in health screening and behaviors [43]. Family support, especially from the mother, played a significant role in promoting BSEP behavior in their daughters. Every month, participants’ feedback through online data helped to understand the effectiveness of the intervention and the role of the mother more clearly.

Understanding BC and screening methods, such as BSE, for early awareness of BC symptoms was crucial. Research has shown that a lack of knowledge about BSEP is one of the reasons why people do not undertake it [47]. The results of the study were consistent with previous research showing that educational interventions that increase BSE knowledge through lectures, discussions, video sharing, or interactive demonstrations were effective in improving adult women’s BSEP [48,49]. CBBHEP was more informative as misperceptions about BC were communicated with facts. Twenty-minute smartphone-based teaching sessions were similarly successful in encouraging BSEP behavior in adult women. Understanding BC (classification, common site, risk factors, and screening measures), BSE methods (procedures, time to do, and observation), and BSEP demonstration comprised the educational intervention. The BSEP score of women who received the educational intervention was higher than that of the control group [29]. WHA, a part of CBBHEP, has an additional feature of making diary notes and sharing feedback to show acceptability and engagement in the intervention for adapting the behavior of BSEP.

Positive behaviors regarding BC screening measures were produced through attitude-focused interventions. By providing information about facts, at-risk groups, and the significance of screening procedures, CBBHEP also incorporated actions to foster a positive attitude toward BSEP. Additional conversations on their concerns about BC and BSE were held with the participants and their mothers in small groups of five to seven people. Women’s health apps received similar information. It was discovered that an educational intervention that encouraged a positive attitude toward BSEP worked well. Face-to-face group discussions during the educational interventions revealed a favorable attitude toward BSE, demonstrating a substantial increase in their views regarding BSEP following the group intervention [26,50]. CBBHEP was different as a positive attitude was enhanced through not only group discussion but also mother support.

Self-efficacy in conducting BSE was also assessed using a self-reported tool and at each follow-up session, including the fourth, eighth, and twelfth weeks of the intervention process. After the intervention, it consistently increased compared to the baseline. The program’s activities are comparable to those found in the literature that support the enhancement of self-efficacy. A health education intervention has been implemented to enhance women’s BSE performance. The intervention consisted of several techniques to boost women’s self-efficacy, such as verbal encouragement, group support, studying the educator’s conduct, and determining whether it is feasible. The self-efficacy and BSEP of the educational group were considerably greater than those of the control group [50]. Following the interactive in-person and online interventions, BSE self-efficacy levels increased significantly. The most significant predictor of behavioral change is self-efficacy [46]. CBBHEP was also effective in promoting self-efficacy with the addition of mother support, who continually encourages her daughters to perform BSE and teaches the steps to other daughters as well.

According to the study’s findings, a CBBHEP in the clinical practice of nurses could benefit from incorporating SCT into the concept of cultural identity. The program improved women’s knowledge, attitude, and self-efficacy for BSEP in their cultural context and values, which was crucial from a clinical standpoint for all aged women to be aware of BC and self-conscious of their breast health. Health education and family support, particularly from mothers, could raise BSEP to make it an alternative and successful approach to breast health awareness and early identification of BC symptoms. Mobile-based health education programs are the most popular and successful, and nurse-delivered interventions are required to apply these programs to high-risk women in culturally isolated communities by incorporating their culture. Future study could be conducted at multiple sites and in different communities for intervention effectiveness.

### Limitations

The study’s shortcomings include that it was conducted at a single site to explore phenomena further, and the program needed to be conducted in more settings. Second, some participants delayed the timely submission of their input to the WHAs, which may have been caused by internet problems or a lack of funds to purchase internet packages. To further understand the barriers and pinpoint contextual elements linked to future long-term preventive behavior, qualitative research should be planned.

### Contributions of the Study

The study made contributions to women’s breast health awareness in the following ways,

1. The study created public awareness about negative cultural beliefs related to BC and BSE, such as taboo, to touch oneself, and making positive perceptions about body parts, such as a female body part, which is also a part of health, not only a part of sexuality.
2. This study created public awareness that BC can be treated if diagnosed at an early stage; early diagnosis increases the survival rate of patients. This study created public awareness about BSE as a cost-effective screening measure for self-awareness and early diagnosis of BC at a very early stage.
3. The study created confidence in women and made them responsible for their breast health, such as self-awareness, by reducing their shyness in performing BSE.
4. The study created empowerment among women and encouraged them to talk about BC with their family members, such as with their mothers; it is not related to any wrong concept; it is a part of their body for which they have a responsibility to be aware of.

### Conclusions

The BSEP in high-risk women was significantly affected by the CBBHEP as an intervention. At the 12-week follow-up, the BSEP results were significant, indicating that the intervention had an impact. The promotion of BSEP behavior was significantly impacted by family support, particularly from mothers. Nurses in comparable faith-based communities can perform such interventions.

## Supplementary material

10.2196/85340 Checklist 1 CONSORT checklist.

## Abbreviations

ANCOVA: analysis of covariance
BC: breast cancer
BSEP: breast self-examination practice
CBBHEP: culture-based breast health education program
CONSORT: Consolidated Standards of Reporting Trials
mHealth: mobile health
SCT: social cognitive theory
WHA: Women’s Health app
